# Increased matrix metalloproteinase-1 expression by coexposure to UVA and cigarette sidestream smoke and contribution of histone acetylation

**DOI:** 10.1186/s41021-025-00325-z

**Published:** 2025-01-26

**Authors:** Ryoma Ito, Yukako Komaki, Yuko Ibuki

**Affiliations:** Graduate Division of Nutritional and Environmental Sciences, University of Shizuoka, Yada 52- 1, Suruga-ku, Shizuoka, 422-8526 Japan

**Keywords:** Cigarette sidestream smoke, UVA, MMP-1, COL1A1, Histone, Acetylation, Aging

## Abstract

**Background:**

Skin is exposed to various environmental factors throughout life, and some of these factors are known to contribute to skin aging. Long-term solar UV exposure is a well-known cause of skin aging, as is cigarette smoke, which contains a number of chemicals. In this study, combined effect of UVA and cigarette sidestream smoke (CSS) on matrix metalloproteinase-1 (MMP-1) induction was investigated. MMP-1 is the main protease that initiates collagen type I fiber fragmentation in human skin and is associated with aging.

**Results:**

Combined exposure to UVA and CSS enhanced MMP-1 induction, accompanied by collagen type I (*COL1A1*) gene suppression. The basal expression of MMP-1 was higher in senescent cells than in normal cells, with a pronounced increase after coexposure to UVA and CSS. UVA irradiation resulted in global histone H3 acetylation, and we considered this was responsible for the MMP-1 upregulation. Histone deacetylase inhibitors, sodium acetate, propionate, and butyrate, all enhanced the CSS-induced MMP-1 according to the degree of histone acetylation.

**Conclusion:**

These results suggest that UVA and CSS additively induce MMP-1, which may lead to skin aging, and that such combined effect may further promote aging in aged skin. UVA-induced histone acetylation may contribute to MMP-1 induction.

**Supplementary Information:**

The online version contains supplementary material available at 10.1186/s41021-025-00325-z.

## Introduction

The skin is a primary organ that interacts with the environment. It is regularly exposed to various environmental stressors such as solar light and environmental chemicals. Among them, long-term solar UV radiation (UVR) causes photoaging and skin cancer. Photoaging is characterized by skin wrinkling and laxity, largely due to the alteration of dermal connective tissue, including type I collagen fibrils [1]. Matrix-degrading proteolytic enzymes (matrix metalloproteinases, MMPs) degrade the collagen and UVR has been reported to induce MMPs such as MMP-1, -3 and − 9 [2–5].

Solar UVR wavelength is divided into UVA (315–400 nm), UVB (280–315 nm), and UVC (100–280 nm) [6]. The UV component of terrestrial radiation from the midday sun is approximately 95% UVA and 5% UVB. Stratospheric ozone removes the UVC and part of the UVB (< 290 nm) wavelengths from terrestrial radiation. Both UVA and UVB are known to induce the MMPs [4, 7, 8]. Tewari et al. [9] showed that solar UVB exposure is likely the main cause of photoaging by MMP-1 expression. However, it is generally accepted that UVA influences photoaging more than UVB. UVA radiation is 10 to 100 times more abundant in natural sunlight and penetrates deeper into the dermis than UVB radiation [10]. Dermal fibroblasts are well known to express MMP-1 after UVA irradiation [7, 8]. Furthermore, UVA1, long wavelength (340–400 nm) and comprising 75% of UVA, induced dermal MMPs more than UVB [11]. The greater contribution from different spectrum of UVR, UVA or UVB, may depend on the time point after exposure and the depth of the skin, i.e., epidermis or dermis [5, 11].

Environmental stresses other than UVR also accelerate skin aging by MMPs upregulation [12]. Among these, smoking is a well-known factor for wrinkling and premature skin aging [13]. Smoking increases the number of elastic fibers due to the degradation of elastic materials, as it occurs similarly in solar elastosis [14]. Cigarette smoke extract induces *MMPs* and degrades procollagens in fibroblasts obtained from non-smoking healthy individuals [15, 16], similar to UVR as noted above. An in vivo study also showed that topical or intracutaneous treatment with cigarette smoke extract decreased type I collagen in mouse skin [17]. Interestingly, cigarette smoke and UVA additively induced MMP-1 expression [18], suggesting that several environmental chemicals and UVR may interact and accelerate skin aging. However, the detailed mechanism of the increased MMP-1 induction after coexposure to cigarette smoke and UVA is unclear.

MMP genes such as *MMP13*,* MMP12*,* MMP3*, and *MMP1* are clustered at chromosome 11q22.3. UVB irradiation increases the activity of transcription for most MMP genes. This heightened transcription occurs at specific regions called promoters, where histone H3 acetylation has taken place [19]. Histone acetylation is critical in the regulation of DNA transcription and damage repair, and the degree of acetylation is mediated by histone acetyltransferase (HAT) and histone deacetylase (HDAC) [20, 21]. Global hyperacetylation with increased p300 HAT and decreased HDAC1 and sirtuin1 was observed in chronically sun-exposed skin compared to sun-protected area [22]. Histone acetylation caused by p300 HAT after UVR, promoted *MMP-1* gene transcription in dermal fibroblasts [23, 24].

In this study, the combined effect of UVA and cigarette sidestream smoke (CSS) on MMP-1 induction was investigated. UVA and CSS induced MMP-1 independently, and they were additive. Basal MMP-1 expression was high in senescent cells and further promoted by the combined treatment. The induction mechanism was further investigated using HDAC inhibitors. UVA-induced histone acetylation, and hyperacetylation by HDAC inhibitors remarkably enhanced CSS-induced MMP-1. These data provide insight into the importance of histone acetylation in skin aging accelerated by environmental stressor.

## Materials and methods

### Cell culture

Human dermal fibroblasts, ASF-4-1, were obtained from the Japanese Collection of Research Bioresources (JCRB) Cell Bank (Osaka, Japan). ASF-4-1 [JCRB population doubling level (PDL) = 34] was established from skin of the donor at 36 years and 2 months of age [25]. Eagle’s minimal essential medium (EMEM) with 10% fetal bovine serum (FBS, Biosera, UK) was used as the culture medium, and subculturing was performed at a split ratio of 1:4 every week.

Cellular life span was determined based on PDL. To determine PDL, ΔPDL at each passage was obtained from the equation using the number of seeded cells at a passage and the number of collected cells at the next passage. ΔPDL = log_10_(total cells/plated cells) / log_10_ 2.

### Preparation of CSS

Cigarette smoke generated by the natural combustion of five cigarettes was collected in 100 mL of EMEM by bubbling using a dry vacuum pump as described in our previous paper [26]. This EMEM containing smoke extract is herein referred to as ‘CSS (100%)’.

### Combined exposure to UVA and CSS

Immediately before UVA irradiation, the medium was changed to PBS containing calcium and magnesium. A medium change was also performed for non-irradiated cells. ASF-4-1 cells were exposed to UVA, followed by incubation with or without CSS for 24 h. The wavelength characteristic of the UVA lamp (FL20S-BL-B, HITACHI), reconstructed from manufacturing data, is shown in the Supplementary Materials (Supplementary Fig. S1). Approximately 0.5 cm-thick glass plate was mounted 3 cm above the cell culture dishes to remove UVC and shorter UVB wavelengths from UVA radiation as much as possible. Irradiance was measured using a radiometer (Atto Co., Ltd., Tokyo, Japan) with 365 nm detector (Atto Co., Ltd., Tokyo, Japan). The irradiance was 70 kJ/m^2^/h.

### Reverse transcription-quantitative PCR (RT-qPCR)

Cells coexposed to UVA and CSS were harvested and RNA was extracted with NucleoSpin RNA kit (Takara Bio, Shiga, Japan) according to manufacturer’s protocol. RNA concentrations were determined by Nano Drop 2000 (Thermo Fisher Scientific, Waltham, MA) and the 2 ng (/10 µL) of RNA was reverse transcribed to cDNA using PrimeScript^™^ RT reagent kit (Takara Bio). Each PCR was carried out in quadruplicate in a 10 µL volume using SYBR^™^ Green Master Mix (Thermo Fisher Scientific) for 1 min at 95°C for initial denaturing, followed by 40 cycles of 95°C for 15 sec, 60°C for 15 sec and 72°C for 1 min in the Step One 2.3 Real-Time PCR Cycler (Thermo Fisher Scientific). The sequences of the primers used for qPCR are as follows; *MMP-1*: f-5’GGGAGATCATCGGGACAACTC3’, r-5’TGAGCATCCCCTCCAATACC3’, *COL1A1*: f-5’GATTCCCTGGACCTAAAGGTGC3’, r-5’AGCCTCTCCATCTTTGCCAGCA3’, *GAPDH*: f-5’GAGTCAACGATTTGGTCGT3’, r-5’TTGATTTTGGAGGGATCTCG3’. The quantification cycle (CQ) values were used for relative quantitation of samples. Values for each gene were normalized to GAPDH expression levels and expressed as fold increase over untreated control.

### Western blotting

The treated cells were scraped, pelleted, suspended in lysis buffer (100 mM Tris-HCl (pH 8.0), 50 mM EDTA, 0.5% Triton X-100) containing protease inhibitors (Wako #161-26023), sonicated for 30 s, mixed with an equal volume of 2x sample buffer (0.1 M Tris-HCl (pH6.8), 4% SDS, 20% glycerol, 12% 2-mercaptoethanol, 0.004% bromophenol blue), and boiled for 5 min.

Proteins were separated by 10 or 15% polyacrylamide gels and transferred to polyvinylidene fluoride membranes. After blocking with 0.2% skim milk or albumin, membranes were incubated with primary antibodies against global N-terminal acetyl-histone H3 (#06-599), acetyl-histone H3 (Lys 9) (#06-942), acetyl-histone H3 (Lys 14) (#06-911) (Merck Millipore, Burlington, MA), MMP-1 (GTX100534) (GeneTex, Alton Pkwy Irvine, CA), and actin (sc-1615) (Santa Cruz Biotechnology, Dallas, TX) overnight at 4℃, then incubated with secondary antibody conjugated with horseradish peroxidase (Jackson ImmunoResearch Laboratories, West Grove, PA) for 2 h. Protein expression was visualized with an enhanced chemiluminescence plus detection kit (Thermo Fisher Scientific, Waltham, MA).

### Cell survival assay

Treated cells were harvested using trypsin-EDTA solution and then suspended in EMEM. Trypan blue solution (0.3%) was added to the cell suspension (1:1) for the discrimination of dead cells. More than 400 cells per sample were counted under the microscope.

### Statistical analysis

All experiments were repeated two to three times. Data are presented as the mean ± S.D. Data were analyzed by Student’s *t*-test for comparisons between groups.

## Results

### MMP-1 expression after exposure to UVA or CSS

**Fig. 1:**
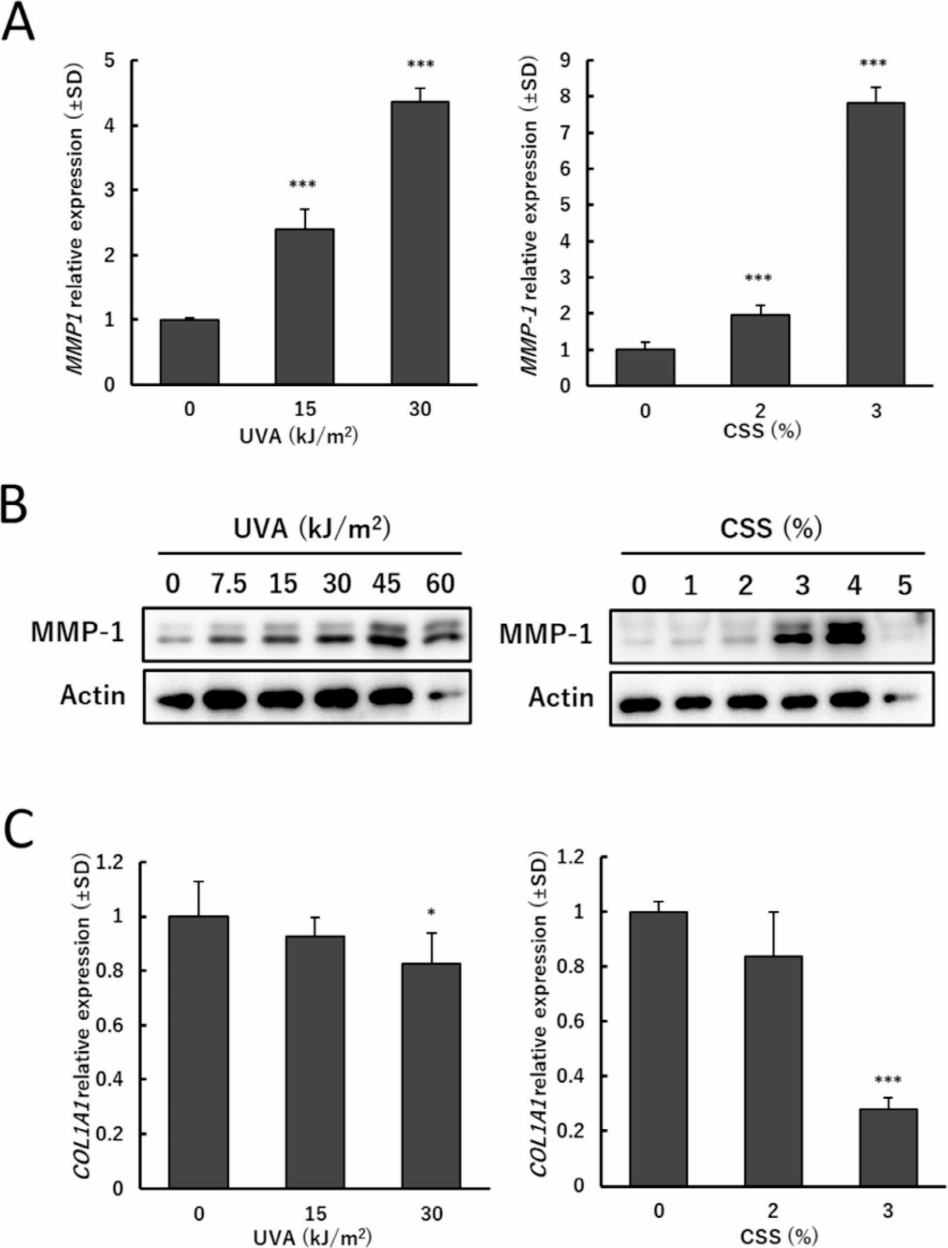
MMP-1 induction after exposure to UVA or CSS. (**A**) (**C**) *MMP-1* and *COL1A1* mRNA expression 24 h after exposure to UVA or CSS. ASF-4-1 cells were irradiated with UVA (15 or 30 kJ/m^2^) or treated with CSS (2 or 3%). After incubation for 24 h, RT-qPCR was performed as described in Materials and Methods. (**B**) Protein levels of MMP-1 24 h after exposure to UVA or CSS. ASF-4-1 cells were irradiated with UVA (up to 60 kJ/m^2^) or treated with CSS (up to 5%). After incubation for 24 h, cells were lysed and Western blotting was performed as described in Materials and Methods. Actin was used as a standard for the equal loading of proteins for SDS–PAGE. *p < 0.05. ***p < 0.001 (vs. untreated (UVA 0 kJ/m^2^ or CSS 0%))

The ASF-4-1 cell line, established from donor skin at 36 years and 2 months of age [25], has a finite lifespan and was used between PDL35-47. UVA exposure significantly induced *MMP-1* mRNA (Fig. 1A). Treatment with CSS above 2% also promoted *MMP1* mRNA. As CSS above 5% showed drastic cell death (data not shown), we mainly used CSS below 4% in this study. Protein levels yielded similar results, i.e., MMP-1 was induced in a UVA- and CSS-dose-dependent manner (Fig. 1B). Since 5% of CSS caused 100% of cell death after treatment for 24 h, the decrease of MMP-1 was probably due to cytotoxicity. *COL1A1* encodes the major component of type I collagen that can be degraded by MMP-1. *COL1A1* mRNA was significantly downregulated by both UVA and CSS (Fig. 1C). These results were consistent with the previous reports on MMP-1 induction [7, 8, 15] and collagen reduction [15–17] after treatment with UVA or cigarette mainstream smoke, while this study used CSS.

### Combined exposure enhanced MMP-1 expression

**Fig. 2:**
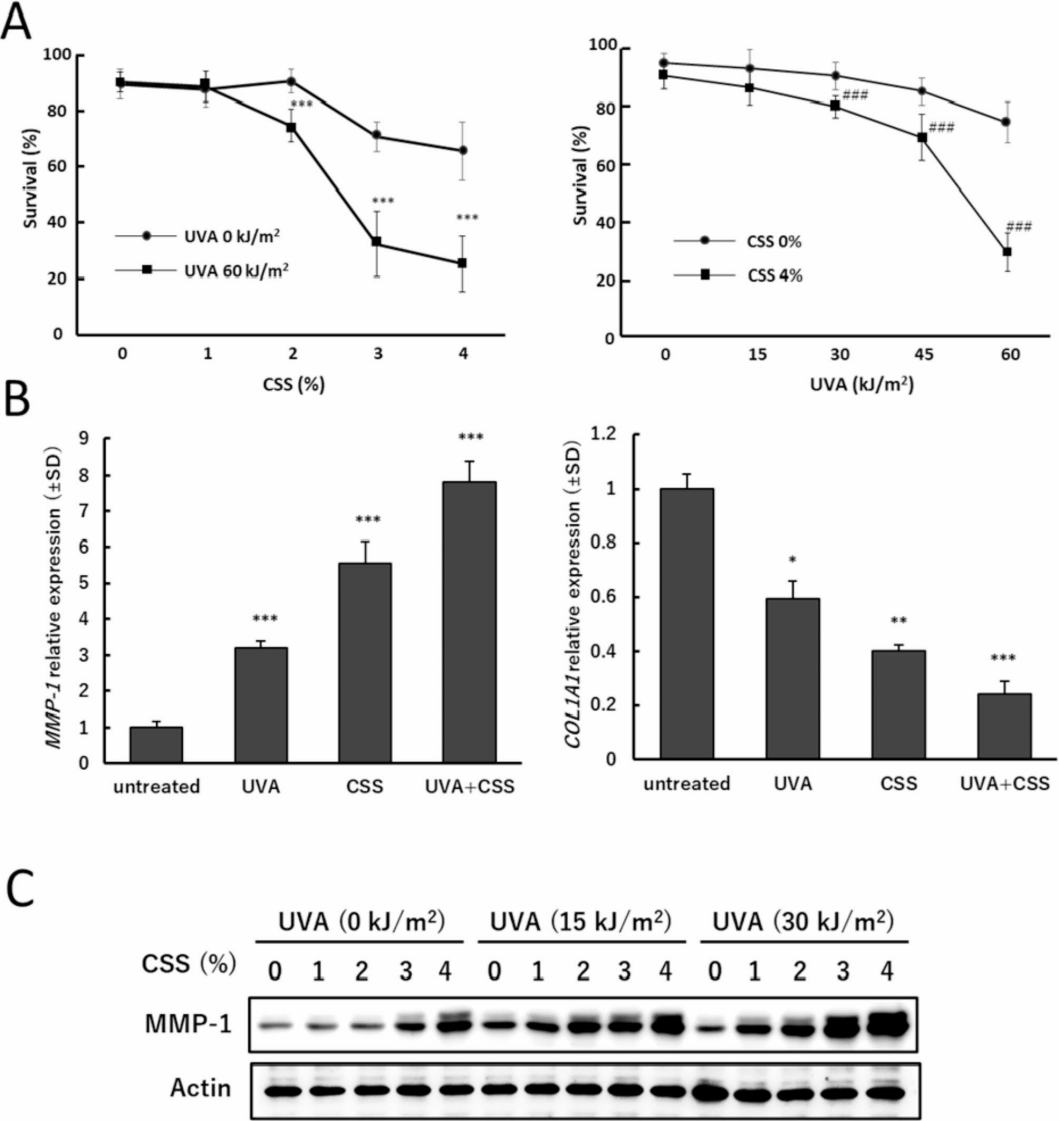
Cell survival and MMP-1 induction after combined exposure to UVA and CSS. (**A**) Survival after coexposure to UVA and CSS. ASF-4-1 cells were irradiated with UVA (up to 60 kJ/m^2^), followed by treatment with CSS (up to 4%). After incubation for 24 h, cell survival was determined using trypan blue assay as described in Materials and Methods. ***p < 0.001 (vs. UVA 0 kJ/m^2^), ^###^*p* < 0.001 (vs. CSS 0%). (**B**) *MMP-1* and *COL1A1* mRNA expression 24 h after combined exposure to UVA and CSS. ASF-4-1 cells were irradiated with UVA (15 kJ/m^2^), followed by treatment with CSS (2%). After incubation for 24 h, RT-qPCR was performed. *p < 0.05, **p < 0.01, ***p < 0.001 (vs. untreated). (**C**) MMP-1 protein levels 24 h after combined exposure to UVA and CSS. ASF-4-1 cells were irradiated with UVA (15 or 30 kJ/m^2^), followed by treatment with CSS (up to 4%). After incubation for 24 h, cells were lysed and Western blotting was performed. Actin was used as a standard for the equal loading of proteins for SDS–PAGE

Combined exposure to UVA and CSS synergistically enhanced cell death (Fig. 2A). Treatment with CSS up to 4% for 24 h showed approximately 20 to 30% cell death, which increased to 70 to 80% with UVA pre-irradiation. Similarly, the slight cell death after UVA irradiation was significantly enhanced after CSS treatment.

Both *MMP-1* and *COL1A1* mRNA were additively altered after combined treatments with UVA and CSS (Fig. 2B). Such additive effect of UVA and CSS on *COL1A1* mRNA suppression has not been reported elsewhere. MMP-1 protein levels were also altered, with CSS-dependent MMP-1 increase significantly enhanced by UVA pre-irradiation (Fig. 2C and Supplementary Fig. S2).

### Cell senescence promoted MMP-1 expression after combined exposure

**Fig. 3:**
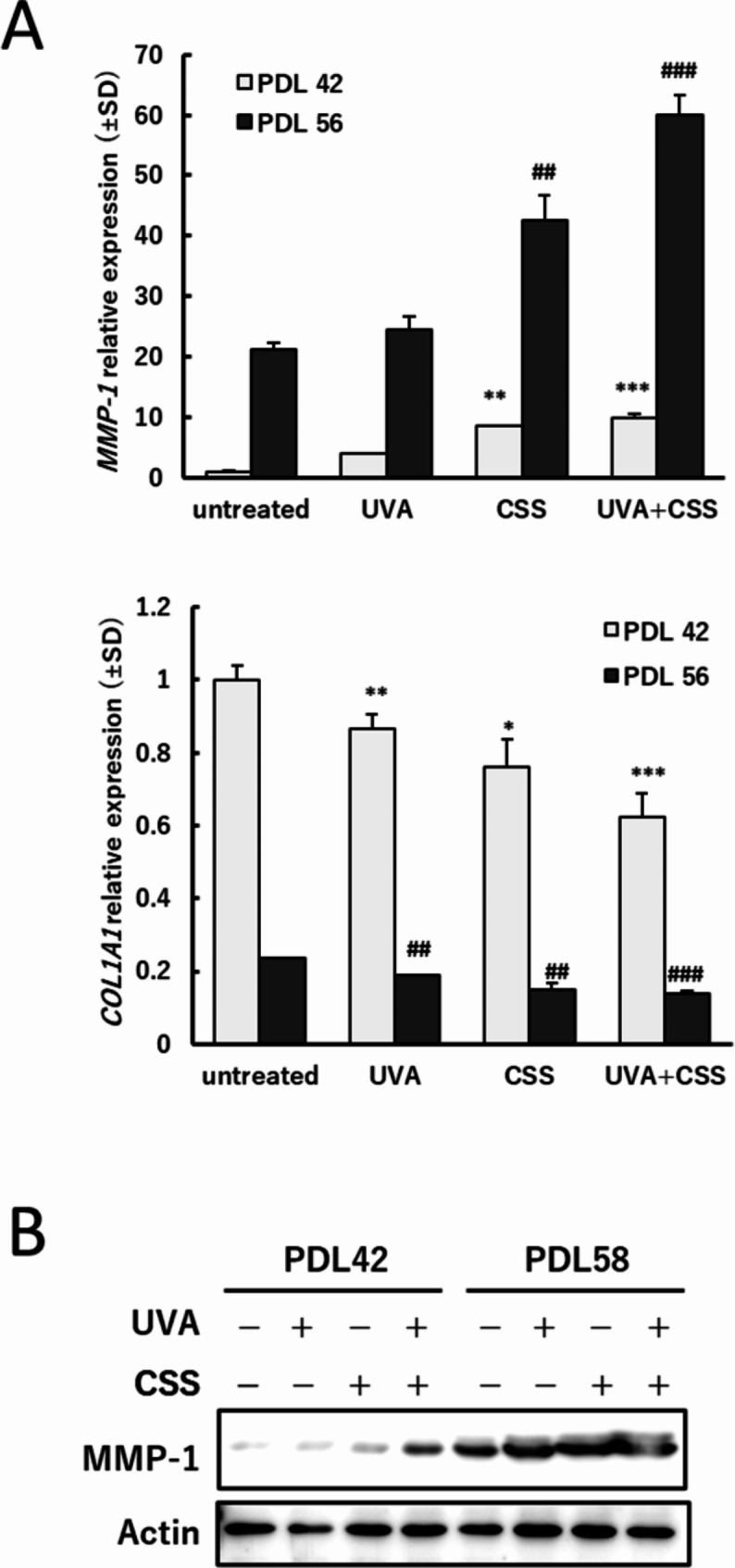
MMP-1 induction in senescent cells after combined exposure to UVA and CSS. ASF-4-1 cells were passaged 1:4 once per week, increasing PDL by 2. Cells above PDL55 exhibit aging phenotype. (**A**) *MMP-1* and *COL1A1* mRNA expression 24 h after combined exposure to UVA and CSS. ASF-4-1 cells with PDL42 (normal cells) and 56 (senescent cells) were irradiated with UVA (15 kJ/m^2^), followed by treatment with CSS (2%). After incubation for 24 h, RT-qPCR was performed. *p < 0.05, **^, ##^*p* < 0.01, ***^, ###^*p* < 0.001 (vs. untreated). (**B**) Protein levels of MMP-1 24 h after combined exposure to UVA and CSS. ASF-4-1 cells (PDL42 and PDL58) were irradiated with UVA (15 kJ/m^2^), followed by treatment with CSS (2%). After incubation for 24 h, cells were lysed and Western blotting was performed. Actin was used as a standard for the equal loading of proteins for SDS–PAGE

The ASF-4-1 cell line is a model system for cellular aging [27]. In this study, the cell passage was repeated and the high PDL cells (PDL56 or 58) were established. From PDL55, remarkably elevated cellular senescence markers for β-galactosidase activity and decreased cell proliferation were marked [28]. The senescent cells (PDL 56) yielded approximately 20 times higher basal *MMP-1* expression than normal cells (PDL 42) (Fig. 3A). Combined exposure to UVA and CSS further augmented the *MMP-1* expression enhanced by cellular senescence. As *COL1A1* mRNA expression in PDL56 cells reduced to 20% of normal cells, the further reduction by combined exposure looks small but was significant. The MMP-1 protein levels were also in proportion to the induction of the mRNA, and UVA-, CSS- and coexposure-induced MMP-1 were promoted in senescent cells (Fig. 3B and Supplementary Fig. S3).

### Histone acetylation augmented MMP-1 expression

**Fig. 4:**
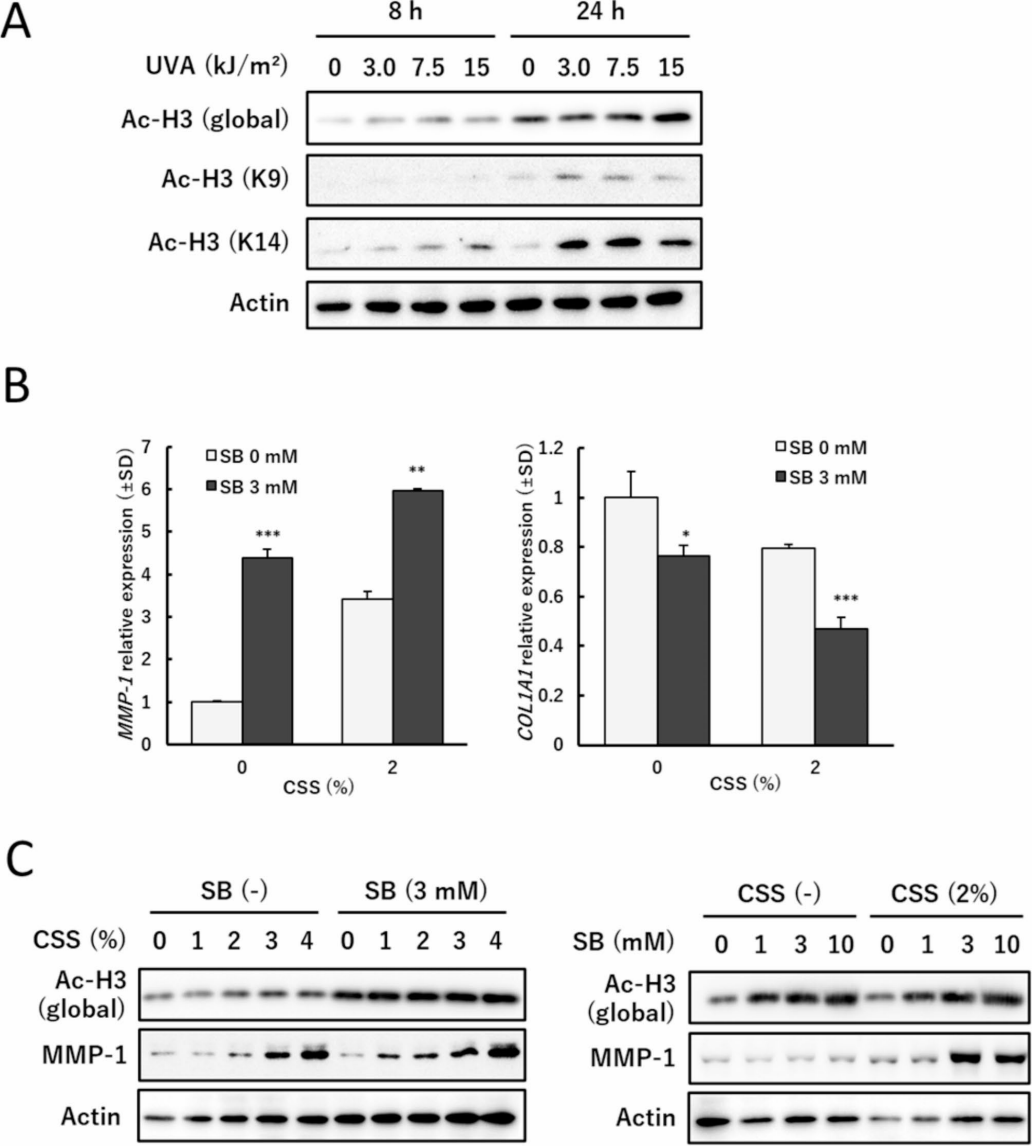
Histone acetylation by UVA or SB, and MMP-1 induction after treatment with CSS. (**A**) Histone acetylation after UVA irradiation. ASF-4-1 cells were irradiated with UVA (up to 15 kJ/m^2^) and cultured for 8–24 h. Western blotting was performed and histone acetylation was analyzed using three types of histone acetylation antibodies. Actin was used as a standard for the equal loading of proteins for SDS–PAGE. (**B**) *MMP-1* and *COL1A1* mRNA expression 24 h after combined exposure to SB and CSS. ASF-4-1 cells were treated with SB (3 mM), followed by treatment with CSS (2%). After incubation for 24 h, RT-qPCR was performed. *p < 0.05, **p < 0.01, ***p < 0.001 (vs. SB 0 mM). (**C**) Protein levels of global histone acetylation and MMP-1 24 h after combined exposure to SB and CSS. ASF-4-1 cells were treated with SB (up to 10 mM) for 24 h, followed by treatment with CSS (up to 4%). After incubation for 24 h, cells were lysed and western blotting was performed

UVR is reported to acetylate histone via the histone acetyltransferase activation, leading to MMP-1 expression [23, 24]. UVA exposure promoted global histone H3 acetylation in a dose dependent manner (Fig. 4A). To further clarify the relationships between histone acetylation and CSS-amplified MMP-1, sodium butyrate (SB), a representative HDAC inhibitor, was dosed instead of UVA exposure, followed by CSS treatment. The *MMP-1* mRNA was remarkably induced by SB, which was further enhanced by CSS (Fig. 4B). The relative decrease was found in *COL1A1*. Figure 4C shows the protein levels after combined treatment with SB and CSS. SB clearly acetylated histone H3 dose-dependently. MMP-1 expression was similar or slightly increased at 24 h in the presence of SB only and enhanced by combined treatment with CSS.

**Fig. 5:**
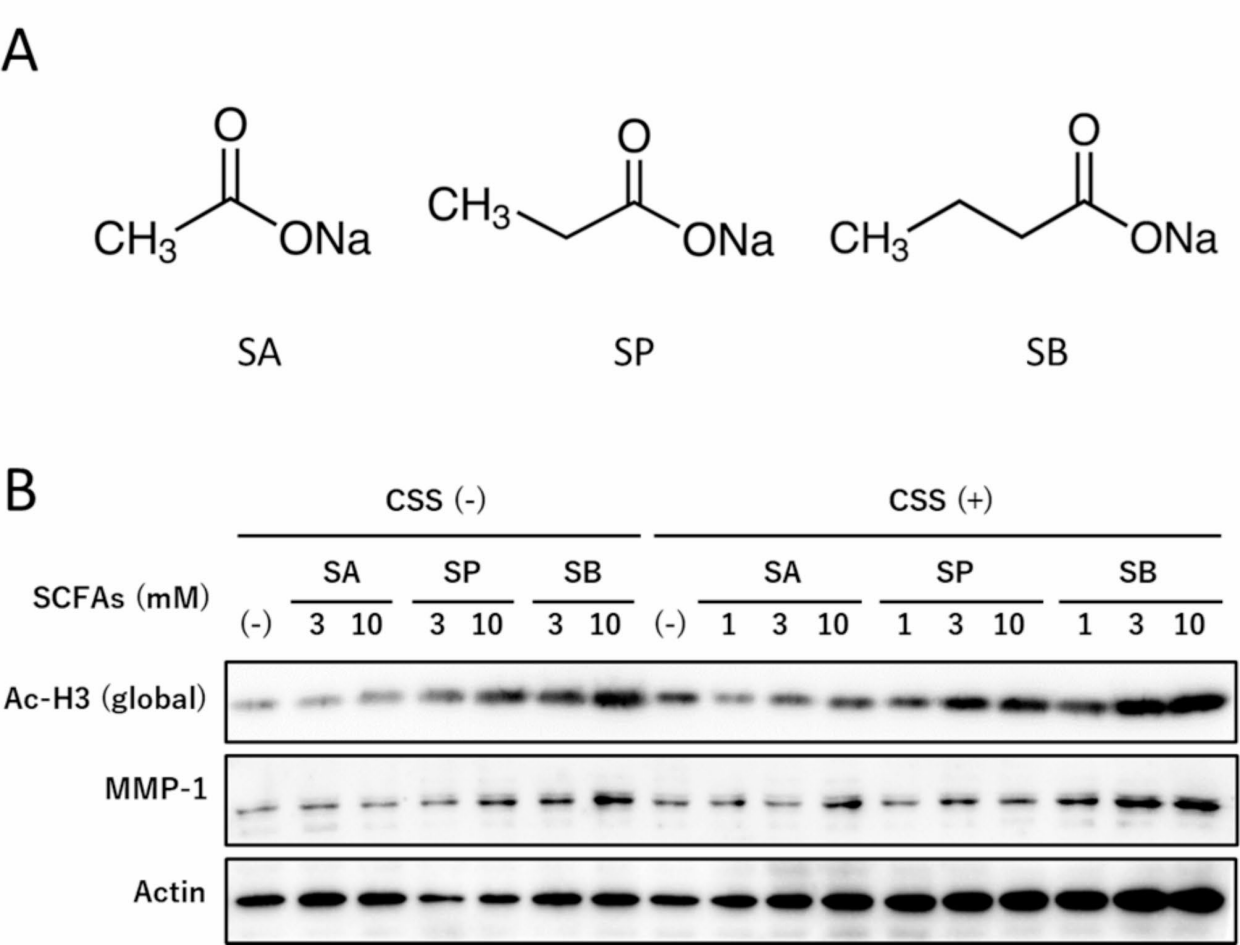
Histone acetylation by SCFAs and MMP-1 induction after treatment with CSS. (**A**) Structure of SCFAs. (**B**) MMP-1 induction and acetylation of histone H3 24 h after combined exposure to SCFAs and CSS. ASF-4-1 cells were treated with SCFAs (up to 10 mM), followed by treatment with CSS (2%). After incubation for 24 h, Western blotting was performed. Actin was used as a standard for the equal loading of proteins for SDS–PAGE

The degree of histone acetylation influenced CSS-induced MMP-1 expression. Other members of short chain fatty acids (SCFAs), sodium acetate (SA) and sodium propionate (SP) were compared with SB (Fig. 5 and Supplementary Fig. S4). Histone acetylation increased in the order of SCFA chain lengths, SA < SP < SB. The MMP-1 induction was proportional to the acetylation level. SA, with the lowest acetylation, only slightly increased MMP-1 after combined exposure, whereas SB, with the strongest effect, showed a very pronounced induction.

## Discussion

Collagen fibrils are largely responsible for skin strength and resilience, and the degradation and the alteration in structure link to skin aging including skin wrinkling and laxity [1]. Type I collagen is the major dermal extracellular matrix structural protein, and its precursor molecule, procollagen, is synthesized by dermal fibroblasts [29]. UVR is a typical cause for skin aging, and is known to alter dermal collagen by two pathways, collagen degradation and inhibition of procollagen biosynthesis. This study showed that UVA irradiation activated both pathways via MMP-1 induction and COL1A1 suppression, which was similarly observed in CSS treatment and exacerbated by combined treatment.

Cellular oxidation is a major contributing factor to MMP-1 induction [12]. Reactive oxygen species (ROS) including singlet oxygen produced after UVA exposure elevate MMP-1 [30]. We previously showed that CSS also increases intracellular ROS [26]. CSS-induced lipid peroxidation and subsequent production of 4-hydroxynonenal is also reported to generate ROS [31]. ROS activate the mitogen-activated protein kinase (MAPK) family, composed of extracellular signal-regulated kinase (ERK), c-Jun NH2-terminal kinase (JNK) and p38. This family is crucial for activating c-Fos and c-Jun, which combine to form the transcriptional factor AP-1, essential in *MMPs* transcription. In our preliminary experiments, the specific inhibitors for MAPKs, U0126 for ERK and SP600125 for JNK, effectively suppressed the expression of MMP-1 after coexposure to UVA and CSS (data not shown), suggesting that both UVA and CSS activate MAPKs, leading to MMP induction. Furthermore, AP-1 is known to inhibit transforming growth factor-β (TGF-β), an important regulator of procollagen type I production. UVR downregulated TGF-β receptor signaling [32] and UVA reduced TGF-β-induced production of collagen [33]. In addition, TGF-β inhibited CSS-induced *MMP-1* mRNA [34]. TGF-β signaling may be involved in our finding of an enhanced decrease in *COL1A1* after co-exposure to UVA and CSS. These complicated pathways arising from ROS would alter the collagen conditions related to skin aging. On the other hand, there are reports supporting that UVB-induced DNA damage triggers the induction of *MMPs* [35, 36]. MMP-1 induction after UVB irradiation was reduced by treatment with DNA damage repair enzyme [35]. DNA damage activates ATM, which subsequently activates JNK and p38, leading to SP1 binding to the promoter regions of *MMP-2* and *MMP-11* to promote their transcription [36]. Although we tried to eliminate the UVC and UVB components from the UVA irradiation using a glass plate but it is possible there was a slight contribution from UVB. Additionally, although UVA and CSS both generate DNA damage, 8-oxo-deoxyguanosine and DNA double strand break, respectively [26, 37], whether these DNA damages other than UVB-induced pyrimidine dimers can induce MMPs, are not well examined.

Histone acetylation in promoter region at the MMP gene cluster is required for transcriptional activation of genes on this cluster [19]. UVR-induced histone acetylation at the promoter regions of the MMP-1 gene by p300 HAT binding [23, 24] may be a cause of MMP-1 expression after coexposure to UVA and CSS. We found that UVA acetylated histone H3, whereas CSS did not (data not shown), presumably because the aldehydes in CSS readily react with histone lysine residues and partly block the acetylation reaction. However, it is possible that chemicals other than aldehydes contained in CSS acetylate histones, as we have previously demonstrated [38], and further accelerate the induction of MMP-1 with UVA. Treatment with HDAC inhibitors instead of UVR resulted in global histone acetylation and augmented MMP genes [19]. The degree of histone acetylation by three kinds of SCFAs was relative to the amount of CSS-induced MMP-1 (Fig. 5), suggesting the important role of histone acetylation in additive UVA and CSS effects. Furthermore, SCFAs synergistically enhanced ligand-induced expression of aryl hydrocarbon receptor (AhR)-responsive genes [39]. CSS contains numerous components including polycyclic aromatic hydrocarbons which trigger AhR signaling pathways. AhR knockdown and inhibition studies suggested that AhR pathways are required for CSS-induced MMP-1 induction [40]. UVA-induced histone acetylation may promote the CSS-induced AhR pathway, resulting in MMP-1 expression.

MMP-1, an important player for skin aging, was expressed at higher levels in senescent cells than in normal cells, with coexposure to UVA and CSS further enhancing the expression (Fig. 3). Although we used replicatively senescent fibroblasts, the increased MMP-1 level was similarly confirmed in aged (> 80 years old) human skin in vivo, where c-Jun expression and ROS generation were also detected [41]. In aged mouse skin, environmental stressors including UV and CSS sensitively expressed MMPs [42], consistent with our in vitro experiment using replicatively senesced fibroblasts. UVR-increased histone acetylation and decreased HDAC expression were observed in aged human skin, suggesting that histone acetylation by UVR has an important role in MMP-1 expression [43]. However, in general, global histone acetylation might be low in cultured senescent cells as cell division and related gene expressions are infinitely suppressed. We need further investigation into whether histone modification at MMP promoter regions differs between normal and senescent cells.

## Conclusion

UVA pre-irradiation promoted CSS-induced MMP-1 expression, which was remarkable in senescent cells. UVA-induced histone acetylation may lead to a combined effect on MMP-1 induction. The skin is exposed to a number of environmental stressors on a daily basis and some of them are associated with skin aging [12, 44]. Histone acetylation could be an important factor in accelerating aging in terms of MMPs expression after combined exposure to UV and various environmental chemicals.

## Electronic supplementary material

Below is the link to the electronic supplementary material.

Supplementary Material 1

## Data Availability

No datasets were generated or analysed during the current study.

## Abbreviations

CSS: Cigarette sidestream smoke
COL1A1: Collagen type I
EMEM: Eagle’s minimal essential medium
FBS: Fetal bovine serum
HAT: Histone acetyltransferase
HDAC: Histone deacetylase
MMPs: Matrix metalloproteinases
PDL: Population doubling level
SA: Sodium acetate
SB: Sodium butyrate
SCFAs: Short chain fatty acids
SP: Sodium propionate
UVR: UV radiation
