# Supplementary material for: Increased matrix metalloproteinase-1 expression by coexposure to UVA and cigarette sidestream smoke and contribution of histone acetylation
Source: Genes Environ. 2025 Jan 26;47:2. doi: 10.1186/s41021-025-00325-z (PMC11765920; doi:10.1186/s41021-025-00325-z)

**SUPPLEMENTARY MATERIALS**

**Increased matrix metalloproteinase-1 expression by coexposure to UVA and cigarette sidestream smoke and contribution of histone acetylation**

**Ryoma Ito^a^, Yukako Komaki^a^, Yuko Ibuki^a*^**

**^a^***Graduate Division of Nutritional and Environmental Sciences, University of Shizuoka, Yada 52-1, Suruga-ku, Shizuoka, 422-8526, Japan*

* Corresponding author e-mail: [ibuki@u-shizuoka-ken.ac.jp](mailto:ibuki@u-shizuoka-ken.ac.jp) (Yuko Ibuki)

**Supplementary Figure S1**

**UVA wavelength characteristic.**

The wavelength characteristic of UVA reconstructed from manufacturer’s product data.

**
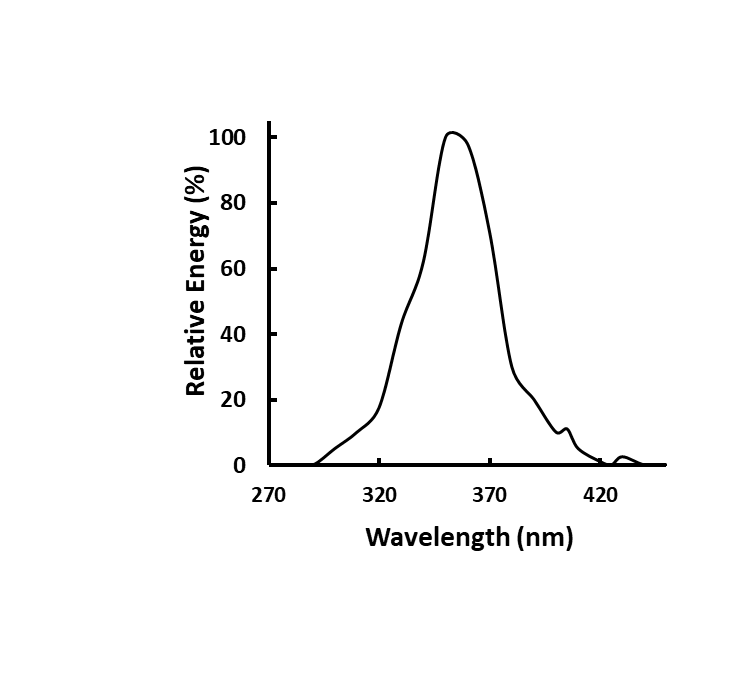
**

**Supplementary Figure S2**

**MMP-1 induction after combined exposure to UVA and CSS.**

The induction of MMP-1 was analyzed by western blotting (Fig. 2C). The intensity of the bands was quantified using Image J software ver. 1.52a and normalized to Actin bands. The ratios to untreated sample (UVA(-) and CSS 0%) are shown.


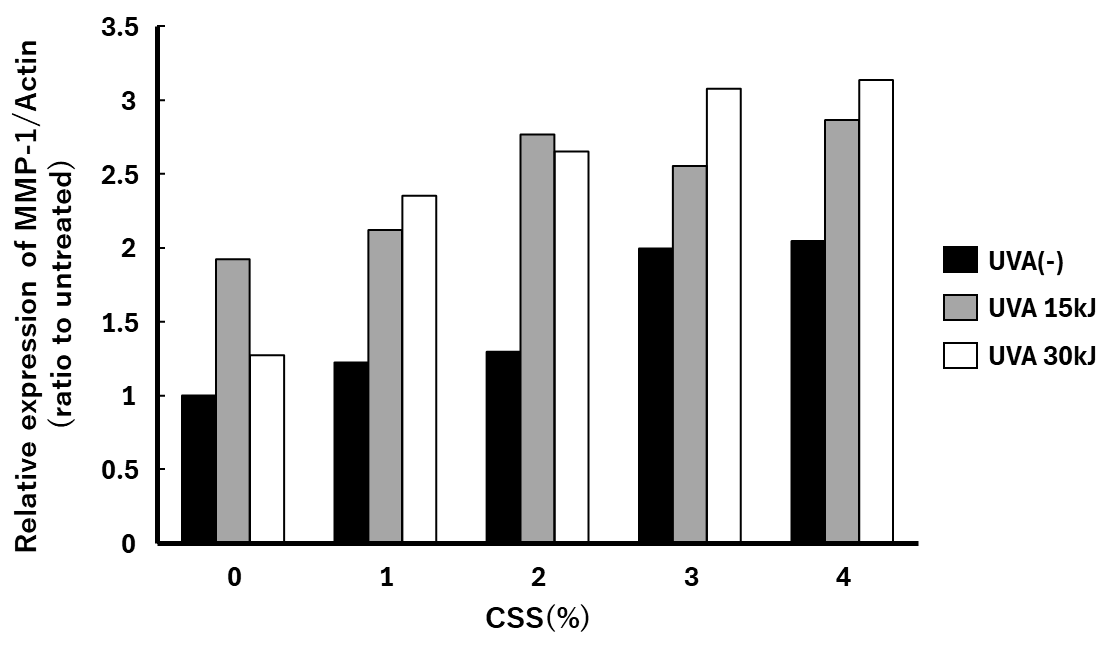


**Supplementary Figure S3**

**MMP-1 induction in senescent cells after combined exposure to UVA and CSS.**

The induction of MMP-1 in normal and senescent cells (PDL42 and 58) was analyzed by western blotting (Fig. 3B). The intensity of the bands was quantified using Image J software ver. 1.52a and normalized to Actin bands. The ratios to untreated sample of PDL 42 (UVA(-) and CSS (-)) are shown.


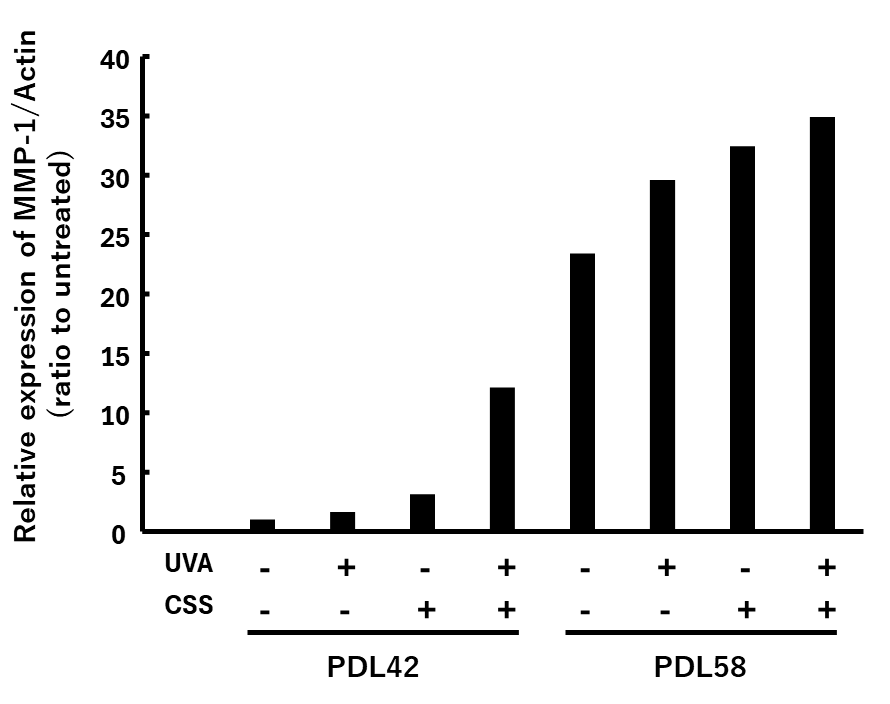


**Supplementary Figure S4**

**MMP-1 induction after combined exposure to SCFAs and CSS.**

The induction of MMP-1 after combined exposure to SCFAs and CSS was analyzed by western blotting (Fig. 5B). The intensity of the bands was quantified using Image J software ver. 1.52a and normalized to Actin bands. The ratios to untreated sample (SCFAs (-) and CSS (-)) are shown.


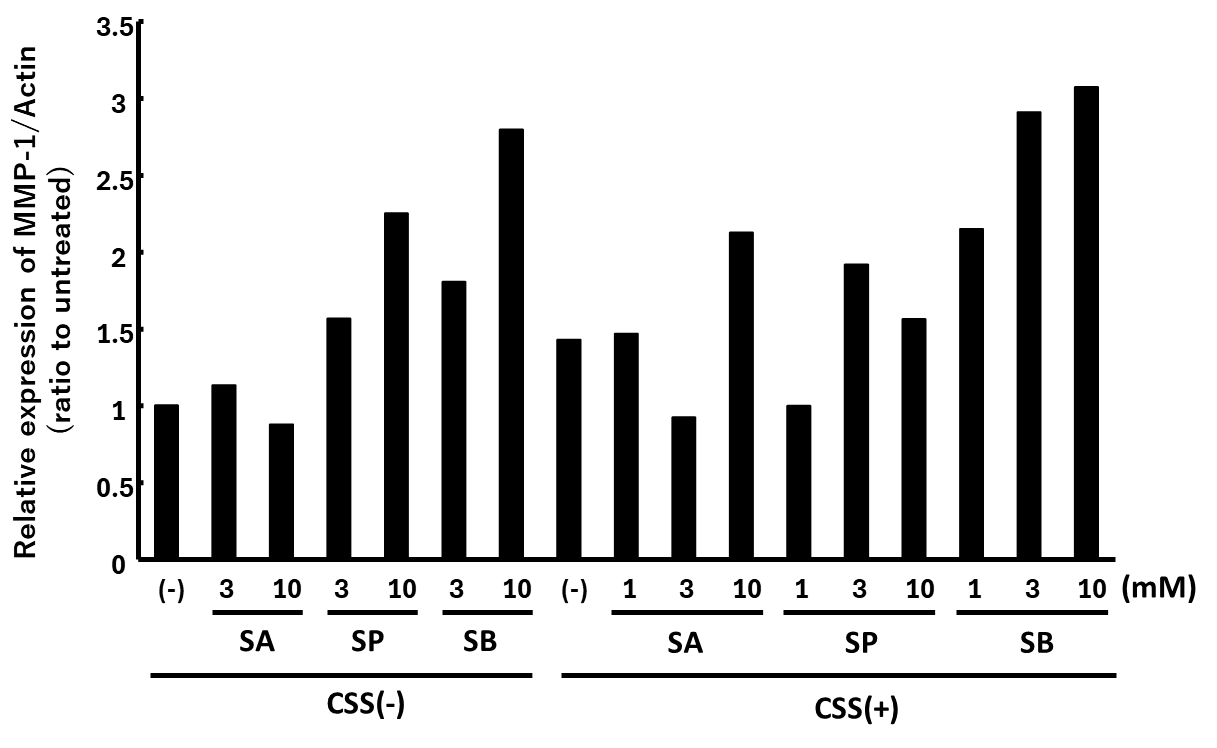

Supplement: Supplementary file 1 — Supplementary Material 1 [file 41021_2025_325_MOESM1_ESM.docx]
